# Health services use among children diagnosed with medium-chain acyl-CoA dehydrogenase deficiency through newborn screening: a cohort study in Ontario, Canada

**DOI:** 10.1186/s13023-019-1001-0

**Published:** 2019-03-22

**Authors:** Maria D. Karaceper, Sara D. Khangura, Kumanan Wilson, Doug Coyle, Marni Brownell, Christine Davies, Linda Dodds, Annette Feigenbaum, Deshayne B. Fell, Scott D. Grosse, Astrid Guttmann, Steven Hawken, Robin Z. Hayeems, Jonathan B. Kronick, Anne-Marie Laberge, Julian Little, Aizeddin Mhanni, John J. Mitchell, Meranda Nakhla, Murray Potter, Chitra Prasad, Cheryl Rockman-Greenberg, Rebecca Sparkes, Sylvia Stockler, Keiko Ueda, Hilary Vallance, Brenda J. Wilson, Pranesh Chakraborty, Beth K. Potter, in collaboration with the Canadian Inherited Metabolic Diseases Research Network (CIMDRN)

**Affiliations:** 1 School of Epidemiology and Public Health, Faculty of Medicine, University of Ottawa, 600 Peter Morand Cr, Ottawa, ON K1G 5Z3 Canada; 2 Ottawa Hospital Research Institute, University of Ottawa, Ottawa, Canada; 3 Manitoba Centre for Health Policy, Department of Community Health Sciences, Max Rady College of Medicine, Rady Faculty of Health Sciences, University of Manitoba, Winnipeg, Canada; 4 Department of Pediatrics, Faculty of Medicine, University of Ottawa, Ottawa, Canada; 5 Newborn Screening Ontario, Children’s Hospital of Eastern Ontario, Ottawa, Canada; 6 Departments of Obstetrics & Gynecology and Pediatrics, Dalhousie University, Halifax, Canada; 7 Department of Pediatrics, Division of Clinical & Metabolic Genetics, The Hospital for Sick Children and University of Toronto, Toronto, Canada; 8 Children’s Hospital of Eastern Ontario Research Institute, Ottawa, Canada; 9 Centers for Disease Control and Prevention, National Center on Birth Defects and Developmental Disabilities, Atlanta, USA; 10 Child Health Evaluative Sciences, The Hospital for Sick Children, Toronto, Canada; 11 ICES, Toronto and Ottawa, Canada; 12 Department of Pediatrics, Division of Paediatric Medicine, The Hospital for Sick Children, University of Toronto, Toronto, Canada; 13 Institute of Health Policy, Management and Evaluation, University of Toronto, Toronto, Canada; 14 Medical Genetics, CHU Sainte-Justine and Department of Pediatrics, Université de Montréal, Montreal, Canada; 15 Department of Paediatrics and Child Health, College of Medicine, Faculty of Health Sciences, University of Manitoba, Winnipeg, Canada; 16 Montreal Children’s Hospital, McGill University, Montreal, Canada; 17 Department of Pathology and Molecular Medicine, Faculty of Health Sciences, McMaster University, Hamilton, Canada; 18 Clinical Genetics Program, McMaster University Medical Centre, Hamilton Health Sciences, Hamilton, Canada; 19 London Health Sciences Centre, Western University, London, Canada; 20 Department of Paediatrics, Section of Clinical Genetics, Alberta Children’s Hospital, Calgary, Canada; 21 Children’s & Women’s Health Centre of British Columbia, Vancouver, Canada; 22 Biochemical Genetics Laboratory, Children’s & Women’s Health Centre of British Columbia, Vancouver, Canada; 23 Department of Pathology, University of British Columbia, Vancouver, Canada; 24 Department of Medicine, Faculty of Medicine, University of Ottawa, Ottawa, Canada; 25 Division of Community Health and Humanities, Memorial University of Newfoundland, St. John’s, Canada

**Keywords:** Newborn screening, Inherited metabolic diseases, Health service utilization, Medium-chain acyl-CoA dehydrogenase deficiency

## Abstract

**Background:**

We describe early health services utilization for children diagnosed with medium-chain acyl-CoA dehydrogenase (MCAD) deficiency through newborn screening in Ontario, Canada, relative to a screen negative comparison cohort.

**Methods:**

Eligible children were identified via newborn screening between April 1, 2006 and March 31, 2010. Age-stratified rates of physician encounters, emergency department (ED) visits and inpatient hospitalizations to March 31, 2012 were compared using incidence rate ratios (IRR) and incidence rate differences (IRD). We used negative binomial regression to adjust IRRs for sex, gestational age, birth weight, socioeconomic status and rural/urban residence.

**Results:**

Throughout the first few years of life, children with MCAD deficiency (*n* = 40) experienced statistically significantly higher rates of physician encounters, ED visits, and hospital stays compared with the screen negative cohort. The highest rates of ED visits and hospitalizations in the MCAD deficiency cohort occurred from 6 months to 2 years of age (ED use: 2.1–2.5 visits per child per year; hospitalization: 0.5–0.6 visits per child per year), after which rates gradually declined.

**Conclusions:**

This study confirms that young children with MCAD deficiency use health services more frequently than the general population throughout the first few years of life. Rates of service use in this population gradually diminish after 24 months of age.

## Background

Medium-chain acyl-CoA dehydrogenase (MCAD) deficiency is a fatty acid beta-oxidation disorder with an estimated birth prevalence of approximately 1:5000 to 1:20,000 in North America and northern Europe [1–4]. Patients are at risk of acute metabolic decompensation during times of physiological stress, such as prolonged fasting and viral illness, with high morbidity and risk of mortality [5, 6]. Early diagnosis is critical because most adverse outcomes are preventable with long-term therapy that includes avoidance of fasting as well as provision of rapidly accessible carbohydrates and close medical monitoring during intercurrent illness [4, 6]. Preventive parenteral glucose is often administered in the emergency department (ED) during high-risk periods. Acute crises require ED management and sometimes inpatient hospitalization.

Timely diagnosis of MCAD deficiency through newborn blood spot screening followed by appropriate management dramatically reduces the risks of acute metabolic crises, early death, and long-term disability, although specific estimates vary across studies [7–10] and some infants with MCAD deficiency experience severe and potentially fatal early neonatal illness prior to the availability of newborn screening results [11]. Multiple economic studies have concluded that newborn screening for MCAD deficiency appears cost-effective [10, 12–17]. A Dutch study calculated that the avoided costs of institutional care for children with neurological disability caused by late-diagnosed MCAD deficiency offset almost half the cost of screening [12]. Similarly, simulations from a cost-effectiveness analysis that incorporated primary data from a US cohort predicted that the majority of the costs associated with newborn screening for MCAD deficiency would be offset by the avoidance of severe adverse outcomes [16].

Screening has also led to a different spectrum of observed cases, with a higher proportion of children with MCAD deficiency identified by newborn screening predicted to have milder forms of disease, relative to clinically identified cases [18–21]. A greater understanding of how MCAD deficiency impacts healthcare utilization among children diagnosed asymptomatically through newborn screening programs can support healthcare providers in communicating with families about the expected clinical course and health service needs of their children.

Newborn Screening Ontario is Canada’s largest newborn screening program and coordinates screening for approximately 140,000 babies born each year, with MCAD deficiency having been added to the screening panel in April, 2006 [1]. The availability of population-based data that captures information about the use of health services for all Ontario residents provides a unique opportunity to investigate healthcare utilization patterns for young children with MCAD deficiency detected by population-based screening relative to young children with negative newborn screening results. We hypothesized that age-stratified rates of healthcare use would be modestly higher among children with MCAD deficiency than among children in a screen-negative comparison cohort over the same time period. Because we had data on outcomes for only those children identified through newborn screening, we were unable to calculate the reduction in healthcare use relative to children with MCAD deficiency born prior to the introduction of expanded newborn screening in Ontario.

## Methods

### Study population and data sources

We initially included all children who were born in Ontario and received newborn screening between April 1, 2006 and March 31, 2010. Individuals were excluded from the study if they were ineligible for public health insurance coverage at the time of birth (e.g., non-residents) or died within 24 h following birth (in Ontario, blood spot samples for newborn screening are considered valid when collected at or later than 24 h of age).

The cohort of children with MCAD deficiency included screen-identified children with a diagnosis confirmed through follow-up evaluation [1]. Ontario newborns who screen positive are referred to one of five regional newborn screening treatment centres based at pediatric tertiary care hospitals. The treatment centre and the infant’s primary healthcare provider collaborate to contact the parents and arrange diagnostic testing, which typically includes plasma acylcarnitine profiling, urine organic acids analysis, and testing for mutations in the *ACADM* gene [1]. Medical staff at Newborn Screening Ontario review and document the diagnostic results reported by the treatment centres. Infants are considered to have a diagnosis of MCAD deficiency if they have a disease-associated genotype (e.g., homozygous or compound heterozygous for the c.985A > G mutation or for any other mutation associated with the disease), and/or persistent abnormal plasma acylcarnitines, and/or hexanoylglycine detected on urine organic acids analysis. The treatment centre is responsible for ongoing follow-up and management for affected children. There is not a provincial treatment protocol for MCAD deficiency management in Ontario and metabolic physicians tailor care based on patient age and disease characteristics as well as sociodemographic factors [22]. Treatment centres typically provide parental and primary care physician education about fasting avoidance, ‘sick day’ protocols for maintaining glucose levels during illness, and recommendations for medical monitoring during illness; the latter may involve telephone or in-clinic care by treatment centre staff and/or emergency department letters to ensure rapid and appropriate care during at-risk periods. The screen-negative comparison cohort included all children with negative newborn screening results for all screened disorders.

Newborn screening short term follow up data were reviewed by Newborn Screening Ontario  medical staff to confirm the final diagnosis, which was linked to the provincial healthcare patient registry at the ICES as well as to administrative databases encompassing health service encounters from April 1, 2006 through March 31, 2012. These datasets were linked using unique encoded identifiers and analyzed at ICES. Physician encounter data were identified using the Ontario Health Insurance Plan (OHIP) Claims Database, which captures services provided by Ontario physicians who bill OHIP on a fee-for-service basis and services provided by other Ontario physicians, dependent on their model of payment [23]. ED visit data were obtained from the Canadian Institute for Health Information (CIHI) National Ambulatory Care Reporting System [24]. Inpatient hospitalization data were retrieved from the CIHI Discharge Abstract Database [25].

### Covariates

Covariates from the newborn hospital record included sex, birth weight, gestational age, and season of birth. Children were grouped into low (< 2500 g) and normal/high (≥ 2500 g) birth weight categories and were dichotomized as preterm (< 37 weeks’ gestation) or term/post-term (≥ 37 weeks). Season of birth was categorized as January–April, May–August, or September–December.

We used neighborhood-level income quintiles as a proxy measure of socioeconomic status, grouping the two lowest and three highest quintiles to define lower and higher socioeconomic status. Quintiles were based on income data from the 2006 Canadian Census at the “dissemination area” level (populations of approximately 400–700 persons), linked to the postal code of a child’s residence at the time of birth [26, 27]. We used the Rurality Index for Ontario (RIO) to assign urban-rural status to each child’s residence at birth [28]. The RIO is based on population size, density, and travel time to high-level healthcare centres; we used a RIO score of ≥40 to define a rural community, corresponding with the cutoff used to establish rural physician eligibility [29].

### Utilization outcomes

Outcomes were health service encounters, including physician encounters, ED visits, and hospitalizations during the study period. If a child had multiple billed procedures on the same day with the same physician, these were considered as one physician encounter. However, if a child saw multiple physicians on the same day, each was considered a separate physician encounter. Physician encounters excluded laboratory billings but included physician-billed encounters that took place in any location, including in-hospital care. Each ED visit or inpatient hospitalization was considered a separate encounter. If an ED visit led to a hospital admission, this would be counted as both an ED encounter and an inpatient hospitalization so that both of these outcomes, which were analyzed separately, would be true reflections of the frequency of use of the respective services.

### Statistical analysis

We separately summed physician encounters, ED visits and hospitalizations for each child. We calculated each child’s length of follow-up as the time between date of birth and the earliest end points among the following: date of death, date of OHIP eligibility loss (mainly related to emigration from Ontario), or the end of study follow-up (i.e., March 31, 2012). Age-stratified rates were calculated to describe healthcare use in each cohort. Incidence rate ratios (IRR) were calculated to compare healthcare use in the MCAD deficiency and screen negative cohorts on a relative scale. Incidence rate differences (IRD) were used to compare the cohorts on an absolute scale (i.e., taking into account the underlying frequency of healthcare visits), in order to provide an estimate of the number of additional visits that parents and providers may expect among children with MCAD deficiency in each age group. Counts of fewer than 6 participants could not be reported in accordance with privacy policies.

Using the Vuong test as a criterion [30], we selected negative binomial regression to calculate IRRs for health service use comparing the MCAD deficiency and screen negative cohorts while adjusting for all covariates (sex, birth weight, gestational age, season of birth, socioeconomic status, rural/urban residence at birth). Influential outlying observations were identified [31, 32] and truncated to the 99th percentile. Models were stratified by age at the time of the visit (< 1 year of age and ≥ 1 year of age). All statistical analyses were performed using SAS® software version 9.3 (SAS Institute, North Carolina, USA).

## Results

### Study population

Forty children were diagnosed with MCAD deficiency through newborn screening during the study period in Ontario and no children who screened negative were later diagnosed with MCAD deficiency (i.e., there were no known missed cases from the screening program). The screen-negative comparison cohort consisted of 545,355 children. Children with MCAD deficiency were more likely to live in rural communities relative to the comparison cohort (Table 1). No other statistically significant differences were observed between cohort characteristics.

**Table 1:** Geographic and sociodemographic characteristics of the study population

	Study Cohort	
	MCAD deficiency, n (%) (n = 40)	Screen negative, n (%) (*n* = 545,355)
Sex		
Male	20 (50.0)	279,638 (51.3)
Female	20 (50.0)	265,717 (48.7)
Season of Birth		
January – April	12 (30.0)	177,918 (32.6)
May – August	16 (40.0)	192,896 (35.4)
Sept. – Dec.	12 (30.0)	174,541 (32.0)
Birth weight^a^		
< 2500 g	< 6 (≤12.5)	33,027 (6.1)
≥ 2500 g	35–40 (≥87.5)	508,466 (93.9)
Gestational age^a^		
< 37 weeks	< 6 (≤12.5)	42,235 (7.9)
≥ 37 weeks	35–40 (≥87.5)	489,232 (92.1)
Socioeconomic status		
‘Lower’	16 (40.0)	232,269 (42.8)
‘Higher’	24 (60.0)	310,003 (57.2)
Urban-rural status^b^		
Rural	6 (15.0)	34,111 (6.3)
Urban	34 (85.0)	505,236 (93.7)

^a^Results are suppressed for cell sizes < 6

^b^*P* < 0.05 for difference in proportion in the MCADD cohort versus the screen negative comparison cohort

### Age-specific rates of healthcare utilization

#### Physician encounters

**Fig. 1:**
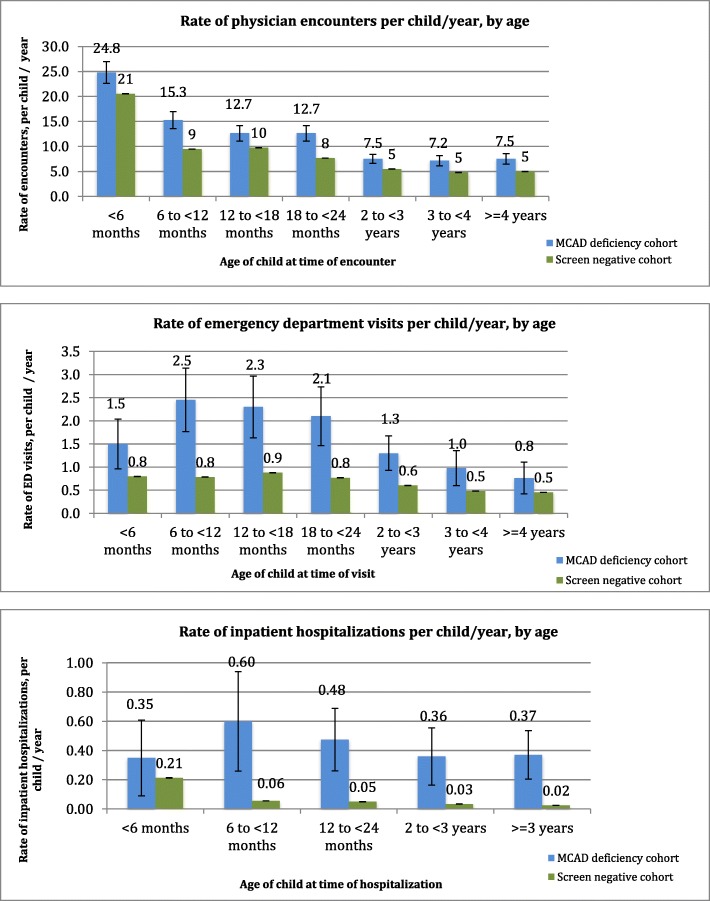
Rates of healthcare encounters by health service type (per child per year)

Relative to other age ranges, children in both cohorts experienced their highest rates of physician encounters from birth to 6 months of age, at 24.8 encounters per child per year (an average of 12.4 encounters per child over the 6 month period) in the MCAD deficiency cohort, and 21 encounters per child per year (10.5 encounters per child over the 6 month period) in the screen negative comparison cohort (Fig. 1). Rates of physician encounters gradually diminished with increasing age in both groups; after age two, rates were 7.2–7.5 encounters per child per year in the MCAD deficiency cohort, and five encounters per child per year in the screen negative cohort. Across all age categories, children with MCAD deficiency experienced statistically significantly higher rates of physician encounters compared to the screen negative group, both on a relative scale, as reflected by the IRR, and on an absolute difference scale, as reflected by the IRD (Table 2). For example, from birth to 6 months of age, children with MCAD deficiency experienced physician encounters approximately 1.2 times more frequently than those with negative newborn screening results (95% confidence interval, 1.1–1.3); on an absolute scale, this reflected approximately 4.2 more encounters per child per year (95% confidence interval, 2.1–6.4), or an average of 2.1 additional physician encounters per child with MCAD deficiency during that 6 month period. Beyond the first 6 months of age, age–specific rates of physician encounters in the MCAD deficiency cohort ranged from 1.3 to 1.7 times higher than the corresponding rates in the screen negative cohort, reflecting an average of 2.0 to 5.8 additional encounters per child per year.

**Table 2:** Age-stratified unadjusted relative (rate ratio) and absolute (rate difference) comparisons of rates of health services encounters in MCAD deficiency cohort versus screen negative cohort

Outcome and age group	Incidence rate ratio (IRR), MCAD deficiency cohort vs. screen negative cohort (95% CI)	Incidence rate difference (IRD), MCAD deficiency cohort vs. screen negative cohort, per child per year (95% CI)
Physician encounters		
< 6 months	1.2 (1.1, 1.3)	4.2 (2.1, 6.4)
6 to < 12 months	1.6 (1.4, 1.8)	5.8 (4.1, 7.5)
12 to < 18 months	1.3 (1.1, 1.5)	2.9 (1.3, 4.5)
18 to < 24 months	1.7 (1.5, 1.9)	5.0 (3.4, 6.6)
2 to < 3 years	1.4 (1.2, 1.5)	2.0 (1.1, 2.9)
3 to < 4 years	1.5 (1.3, 1.7)	2.4 (1.3, 3.4)
≥ 4 years	1.5 (1.3, 1.7)	2.5 (1.4, 3.6)
Emergency department visits		
< 6 months	1.9 (1.3, 2.7)	0.7 (0.2, 1.2)
6 to < 12 months	3.1 (2.4, 4.1)	1.7 (1.0, 2.4)
12 to < 18 months	2.6 (2.0, 3.5)	1.4 (0.8, 2.1)
18 to < 24 months	2.7 (2.0, 3.7)	1.3 (0.7, 2.0)
2 to < 3 years	2.1 (1.6, 2.9)	0.7 (0.3, 1.1)
3 to < 4 years	2.0 (1.4, 3.0)	0.5 (0.1, 0.9)
≥ 4 years	1.7 (1.1, 2.6)	0.3 (−0.03, 0.7)^a^
Inpatient hospitalizations		
< 6 months	1.6 (0.8, 3.5)^a^	0.1 (− 0.1, 0.4)^a^
6 to < 12 months	10.8 (6.1, 18.9)	0.5 (0.2, 0.9)
12 to < 24 months	9.6 (6.1, 15.0)	0.4 (0.2, 0.6)
2 to < 3 years	10.8 (6.3, 18.7)	0.3 (0.1, 0.5)
≥ 3 years	15.2 (9.7, 23.8)	0.4 (0.2, 0.5)

^a^Difference not statistically significant, *p* > 0.05; all other rates were statistically significantly different between the two cohorts

#### ED visits

Children with MCAD deficiency experienced the highest rates of ED visits between the ages of 6 months and 2 years, with the peak rate occurring between six and 12 months, at 2.5 visits per child per year (i.e., approximately 1.3 visits to the ED for each child during that 6 month period) (Fig. 1). The most commonly documented reasons prompting ED visits among children with MCAD deficiency were nausea with vomiting, fever, and cough. In contrast, children in the screen negative cohort experienced a relatively stable rate of ED visits from birth to 2 years of age (0.8–0.9 visits per child per year), with no peak during the six- to 12-month age period. Similar to the cohort of children with MCAD deficiency, in the screen negative cohort, the most commonly documented reasons for ED visits were fever, cough, and nausea with vomiting. Both cohorts experienced gradually lower rates of ED visits after 2 years of age.

On a relative (IRR) scale, children with MCAD deficiency experienced a higher frequency of ED visits compared with the screen negative cohort during the entire follow-up period; the highest IRR, from six to 12 months of age, indicated a visit rate that was 3.1 times higher in the MCAD deficiency cohort (95% confidence interval, 2.4–4.1) (Table 2). On an absolute (IRD) scale, during the highest risk period of six to 12 months of age, children with MCAD deficiency experienced approximately 1.7 additional visits per child per year relative to the screen negative cohort (95% confidence interval, 1.0–2.4), translating to approximately 0.9 additional ED visits per child during that 6 month period. By the time children were 4 years of age or older, the IRR for ED visits was 1.7 for the MCAD deficiency group and was still statistically significant (95% confidence interval, 1.1–2.6), while the IRD reflected 0.3 additional visits per child per year and was not statistically significant (95% confidence interval, − 0.03-0.7).

#### Inpatient hospitalizations

Confidence intervals around the estimated rates of inpatient hospital care for children with MCAD deficiency were wide, reflecting large random variation due to the small numbers of visits in this small cohort (Fig. 1). Aligned with the results for ED visits, children with MCAD deficiency experienced the highest rates of inpatient hospitalization from six to 12 months of age, at 0.6 visits per child per year, or, on average, one hospital admission per three children with MCAD deficiency during that 6 month period. After 12 months of age, rates of hospitalization gradually declined in the MCAD deficiency cohort, to a low of 0.36–0.37 visits per child per year after 2 years of age. Among children with MCAD deficiency, the diagnoses that were most commonly documented in association with inpatient hospital admissions were the underlying fatty acid oxidation disorder and gastroenteritis and colitis. In the screen negative cohort, the rate of inpatient hospitalization was highest from birth to 6 months of age (0.21 visits per child per year, or approximately one visit per every 10 children during that 6 month period), declining to 0.06 visits per child per year from six to 12 months of age and remaining low throughout the remaining age categories. Within the screen negative cohort, the diagnoses most commonly associated with inpatient hospital admissions were neonatal jaundice, pneumonia, and acute bronchiolitis.

Rates of inpatient hospitalization were much higher in the MCAD deficiency cohort relative to the screen negative cohort on a relative (IRR) scale from the age of 6 months onward (Table 2); children with MCAD deficiency experienced a 1.6 times higher hospitalization rate from birth to 6 months of age relative to those with screen negative results (95% confidence interval, 0.8–3.5), but this increased to a 10.8 times higher rate of hospital admission from six to 12 months of age (95% confidence interval, 6.8–18.9) and remained high. However, due to the low frequency of hospitalization in both cohorts, this translated into, at its peak, an extra 0.5 hospital stays per child per year in the MCAD deficiency cohort relative to the screen negative cohort from six to 12 months of age (95% confidence interval, 0.2–0.9) (Table 2). Hospitalization rates were statistically significantly higher in the MCAD deficiency cohort relative to the screen negative cohort at all ages with the exception of the period from birth to 6 months of age, based on both the IRR and IRD.

### Analyses adjusted for covariates

Results adjusted for covariates were similar to the unadjusted results reported above. Among children under 1 year of age, following adjustment for covariates, overall rates for all three types of health services remained significantly higher in children with MCAD deficiency relative to children with negative newborn screening results: physician encounters (adjusted IRR [aIRR]: 1.39 [95% confidence interval, 1.18–1.65]), ED visits (aIRR: 2.41 [95% confidence interval, 1.59–3.73]), and inpatient hospitalizations (aIRR: 2.87 [95% confidence interval, 1.41–5.56]) (Table 3). For children 1 year of age and older, the adjusted IRRs comparing rates of physician encounters and ED visits in children with MCAD deficiency to those among screen-negative children were similar to the findings in younger children: physician encounters (aIRR: 1.51 [95% confidence interval, 1.22–1.88]), ED visits (aIRR: 1.98 [95% confidence interval, 1.39–2.89]). The relative difference in inpatient hospitalization rates between children with MCADD and those with screen negative results was much larger for children greater than or equal to 1 year of age (aIRR: 12.97 [95% confidence interval, 6.96–26.52]) relative to children in the first year of life (Table 3). In both age groups, preterm birth (gestational age < 37 weeks) and low birth weight were statistically significant predictors of higher health services use. Lower socioeconomic status was a predictor of higher rates of ED visits and hospitalization, while rural (vs urban) residence was associated with higher rates of ED use.

**Table 3:** Age-stratified adjusted incidence rate ratios for the three service types, MCAD deficiency cohort versus screen negative comparison cohort

	Adjusted incidence rate ratio (95% CI)					
	< 1 year of age			≥1 year of age		
	Physician visits	ED visits	Hospitalizations	Physician visits	ED visits	Hospitalizations
Cohort						
MCAD deficiency	1.39 (1.18–1.65)	2.41 (1.59–3.73)	2.87 (1.41–5.56)	1.51 (1.22–1.88)	1.98 (1.39–2.89)	12.97 (6.96–26.52)
Screen negative	Reference	Reference	Reference	Reference	Reference	Reference
Sex						
Female	0.92 (0.91–0.92)	0.86 (0.85–0.86)	0.76 (0.75–0.77)	0.91 (0.91–0.92)	0.86 (0.85–0.87)	0.78 (0.77–0.80)
Male	Reference	Reference	Reference	Reference	Reference	Reference
Season of birth						
Jan. – Apr.	Reference	Reference	Reference	Reference	Reference	Reference
May – Aug.	1.01 (1.00–1.01)	1.04 (1.03–1.05)	0.98 (0.96–1.00)	0.96 (0.95–0.96)	0.95 (0.95–0.96)	0.94 (0.92–0.97)
Sept. – Dec.	1.00 (0.99–1.00)	1.02 (1.00–1.03)	1.08 (1.06–1.11)	1.00 (0.99–1.00)	1.00 (0.99–1.01)	1.03 (1.00–1.05)
Birth weight						
< 2500 g	1.75 (1.74–1.77)	1.04 (1.02–1.06)	2.14 (2.08–2.21)	1.13 (1.12–1.14)	1.03 (1.01–1.05)	1.59 (1.51–1.66)
≥ 2500 g	Reference	Reference	Reference	Reference	Reference	Reference
Gestational age						
< 37 weeks	1.57 (1.56–1.58)	1.23 (1.21–1.26)	2.65 (2.57–2.73)	1.09 (1.09–1.10)	1.17 (1.15–1.19)	1.42 (1.36–1.48)
≥ 37 weeks	Reference	Reference	Reference	Reference	Reference	Reference
Socioeconomic status						
‘Lower’	1.00 (1.00–1.00)	1.23 (1.22–1.24)	1.09 (1.07–1.11)	0.96 (0.96–0.97)	1.12 (1.11–1.13)	1.10 (1.08–1.13)
‘Higher’	Reference	Reference	Reference	Reference	Reference	Reference
Urban-rural status						
Rural	0.79 (0.78–0.79)	2.34 (2.30–2.37)	1.16 (1.13–1.20)	0.73 (0.72–0.73)	2.28 (2.25–2.31)	1.15 (1.11–1.20)
Urban	Reference	Reference	Reference	Reference	Reference	Reference

## Discussion

We found that children diagnosed with MCAD deficiency through newborn screening used physician services, ED care, and were hospitalized at significantly higher rates compared to a population-based cohort of children with negative newborn screening results over the first several years of age. Previous studies have found that children diagnosed with fatty acid oxidation disorders experience higher rates of health services use relative to children with other inherited metabolic disorders [33, 34]. Thus, this overall finding was not unexpected. The higher rate of physician encounters from birth to 1 year of age that we observed among children with MCAD deficiency relative to the screen negative cohort might be partially explained by visits required for diagnostic evaluation following a positive newborn screening result. However, the relative rate of physician encounters remained similarly elevated throughout the first 4 years. This can be explained by the fact that young children with MCAD deficiency have short fasting limits and are monitored closely via follow-up visits in the metabolic clinic. ED services and inpatient hospitalizations are necessary for children with MCAD deficiency during times of intercurrent illness or for the prevention or treatment of acute crises.

The relatively high use of services in this population reflects the direct costs of effective disease management. Studies conducted in Australia and the Netherlands that compared healthcare service use and costs for individuals with MCAD deficiency who were born prior to screening and identified clinically with those identified by newborn screening found much lower costs in the newborn screening-identified cohorts [12, 35]. Similarly, a US economic evaluation that incorporated primary data from a chart review into a simulation model predicted far lower costs for the treatment of MCAD deficiency and associated sequelae among children identified by screening [16]. Consequently, it can be presumed that health services use for surviving children with MCAD deficiency would have been even higher than observed if expanded newborn screening had not been introduced in 2006 in Ontario.

To our knowledge this is the first North American study to quantify the frequency and patterns of health services use in young children with MCAD deficiency in comparison with unaffected children (those in the general population who received negative newborn screening results). In addition to the studies described above that compared actual or estimated health services use and costs for children diagnosed with MCAD deficiency clinically versus through newborn screening, additional studies have examined use of hospital and specialist care for children with inherited metabolic disorders overall but have not compared those patterns with use by unaffected children and have not reported use separately for children with MCAD deficiency [33, 34].

These findings are important for understanding the impact of this rare inherited metabolic disease on families, healthcare providers, and systems of care. For example, we found that the highest rates of ED visits and inpatient hospitalizations among children with MCAD deficiency identified through newborn screening occurred from 6 months to 2 years of age, which supports previous evidence documenting the highest risk age groups for metabolic decompensation [3]. Families of children with MCAD deficiency can be reassured that ED visit rates declined after age two, to less than one visit per year on average by age three, corroborating and extending newborn screening long-term follow-up studies [33, 35]. Similarly, absolute rates of hospitalization declined over time, to fewer than 0.4 visits per child per year after age two. Following this cohort as children age into later childhood and eventually adulthood could contribute to our currently limited understanding of the longer term healthcare needs of this population [36, 37].

An important limitation of our study was that our analysis of physician encounters did not allow us to distinguish between outpatient and inpatient physician care, due to incomplete information about the location of care in the OHIP database. Thus, in our study, the physician encounter outcome was conflated with the other two outcomes and does not have a straightforward meaning for families and providers who may associate the concept of physician encounters with outpatient care. The impact of this limitation on our findings is particularly challenging to estimate because in Ontario, pediatric specialists based at the tertiary care hospitals do not bill the public insurance plan (OHIP) on a fee-for-service basis. While such specialists do “shadow bill” for tracking purposes, it may be incomplete and, thus, inpatient physician care is likely partially but not fully included in our physician encounter outcome. While we counted ED visits that led to an inpatient admission as part of both outcomes, this overlap appropriately reflects both concepts of a visit to the ED and a hospital stay. Transfers of care from one hospital to another were not distinguished from other hospital stays and may have resulted in an overestimation of the number of new inpatient admissions. A related limitation is that while our exclusion of laboratory billings from the OHIP database served the purpose of ensuring that our physician encounter outcome was specific to encounters involving interactions between patients and physicians (rather than billings that were specific to laboratory analyses), we are unable to quantify from our study the impact of such services on the health care system.

A limitation of our reliance on routinely-collected healthcare administrative data was our inability to fully characterize important clinical and psychosocial variables that may impact healthcare utilization. Thus, we were unable to definitively distinguish the roles of factors related to disease severity, patient co-morbidity, and/or parental perception of need as possible determinants of care for children with MCAD deficiency. We did gain some insight into factors affecting health care use through the standard administrative data-based documentation of reasons prompting ED visits and diagnoses associated with inpatient stays. Symptoms of acute infectious illnesses were the most commonly documented reasons prompting ED visits among children with MCAD deficiency, which aligned with the reasons for ED visits in the screen negative cohort, and is consistent with the use of ED services during intercurrent illness to prevent or to treat acute metabolic crises. The most common diagnoses associated with inpatient hospital admissions for children with MCAD deficiency were the underlying metabolic disorder, and gastroenteritis and colitis, which is consistent with more severe disease-specific exacerbations prompting hospital stays for children with MCAD deficiency. This contrasts with the diagnoses associated with hospital admissions in the screen negative cohort (i.e., neonatal jaundice and acute respiratory infections). A related question not tackled in our research is the impact of geography in relation to access to care; patients who reside near a metabolic centre may experience a different threshold to receive ED care and/or to be admitted to hospital.

A further limitation of our study was that we were unable to assess a possible association of medical encounters with genotype or with other potential indicators of disease severity, for example newborn screening analytes, particularly octanoylcarnitine (C8). While correlations among genotype, biochemical phenotype, and the risk of metabolic crises for MCAD deficiency are not fully established, newborn screening has detected asymptomatic patients with genotypes and/or screening C8 levels that are predictive of milder disease [18, 19, 21, 38]. Metabolic physicians may thus take such indicators into account, particularly when providing guidance to parents about using ED services to prevent crises during minor illnesses.

Studies that link clinical data to healthcare administrative data could help to address these limitations, to better understand how disease severity and the receipt of interventions are associated with health services use, to incorporate additional outcomes, including health economic and patient/family-reported outcomes, and to investigate the nature of health services received in finer detail (e.g., specialist physician and allied health professional care). This is likely to require large collaborative data collection initiatives, such as the US Inborn Errors of Metabolism Information System (IBEM-IS) for newborn screening long-term follow-up (1893 participants enrolled to date at 30 centres across 21 states) [39, 40] and the clinical data component of the Canadian Inherited Metabolic Diseases Research Network (> 700 participants enrolled to date at 13 centres across 7 provinces) [41].

## Conclusions

This study confirms that young children with MCAD deficiency use health services at higher rates relative to children in the general population. However, reassuringly, rates of health service use gradually diminish in this population after 24 months of age. Understanding patterns of health care use for children with conditions targeted by newborn screening can assist families, healthcare providers and policy decision-makers in managing expectations, providing reassurance about prognosis, and informing planning for the needs of affected children.

## Abbreviations

aIRR: adjusted incidence rate ratio
ED: emergency department
IRD: incidence rate difference
IRR: incidence rate ratio
MCAD: medium-chain acyl-CoA dehydrogenase
RIO: Rurality Index for Ontario
